# Volatile Oil of *Acori Graminei Rhizoma*-Induced Apoptosis and Autophagy are dependent on p53 Status in Human Glioma Cells

**DOI:** 10.1038/srep21148

**Published:** 2016-02-19

**Authors:** Lu Chen, Zhuyun Jiang, Hui Ma, Ling Ning, Hongdan Chen, Li Li, Hongyi Qi

**Affiliations:** 1 College of Pharmaceutical Sciences, Southwest University, Chongqing 400715, China; 2 College of Traditional Chinese Medicine, Southwest University, Chongqing 400715, China

## Abstract

*Acori Graminei Rhizoma* is well known for the beneficial effects on CNS disorders in traditional medicine. Though it is frequently prescribed in formulations for brain tumors, the anti-glioma effect has not been examined. We used volatile oil of *Acori Graminei Rhizoma* (VOA) and human glioblastoma multiforme (GBM) cells in this study. We found that VOA exhibited greater growth suppression in p53 wild-type cells than p53 mutant cells and very low effect on fibroblasts and human glial HEB cells. Apoptosis was triggered by VOA with a caspase-dependent way in p53 wild-type A172 cells, while a caspase-independent way in p53 mutant U251 cells. Meanwhile, both A172 and U251 cells treated by VOA displayed autophagic features. Furthermore, p53 decrease was observed along with VOA-induced apoptosis and autophagy in A172 cells. VOA-induced autophagy was mediated through a p53/AMPK/mTOR signaling pathway in A172 cells, while an mTOR-independent signaling pathway in U251 cells. Finally, blockage of autophagy potentiated the proapoptotic effect in both A172 and U251 cells, indicating a protective role of autophagy in VOA-induced cell death. Together, VOA exhibited anti-tumor activity in human GBM cells and induced apoptotic cell death and protective autophagy, which is cell type specific and dependent on p53 status.

Glioblastoma multiforme (GBM) is the most common and aggressive malignant brain tumor. Patients suffering from GBM usually have extremely poor prognosis and the median survival is only 14 months^1^,^2^,^3^,^4^. Chemotherapy is preferably for brain cancer as malignant brain tumor cells may spread and grow within normal tissues, which limits the application of surgery^5^. However, the clinical effectiveness of the current chemotherapeutic agents is often restricted due to the blood-brain barrier (BBB), drug resistance and toxicity^6^,^7^,^8^. Therefore, the continued intensive investigation of new and innovative treatment strategies is urgently needed.

Substantial evidence has demonstrated that natural products-derived extracts and compounds can suppress tumor development and decrease the incidence and severity of cancer in human^9^. Induction of apoptosis is a classical way for natural products to exert their anti-tumor effect. Apoptosis is known as type I programmed cell death and generally characterized by distinct morphological characteristics and energy-dependent biochemical mechanisms. It is well known that the caspase family in many cases involved in the apoptotic cell death, including mitochondria (caspase 9-caspase 3) way and death receptor (caspase 8-caspase 3) way. Furthermore, it has also been recently reported that many natural products participate in killing cancer cells by triggering autophagy, also called type II programmed cell death^10^. Autophagy is an evolutionarily cellular degradation process that involving in the delivery of cytoplasmic materials, such as aged proteins, mis-folded proteins or damaged organelles, for lysosome-dependent degradation following sequestration in double-membrane vesicle (autophagosomes)^11^,^12^. In addition, natural products may also result in a protective autophagy during induction of cancer cell death, which is a process naturally regarded as cellular stress clearance^10^. Apoptosis and autophagy may be interacted with each other and regulated by some common proteins, such as atg5, Bcl-2 and p53. For instance, p53, a tumor suppressor, is well known for coordinating apoptosis to preserve genomic stability and prevent tumor formation. Recently reports have also suggested the involvement of p53 in the autophagic pathway.

*Acori Graminei Rhizoma*, the dry rhizome of acorusgramineus solander (Araceae), has been used in Chinese medicine for more than hundreds of years. Traditionally, *Acori Graminei Rhizoma* is mainly used for central nervous system (CNS) disorders. Investigations in the past decade mainly focused on its action in ameliorating learning and memory deficits and improving the cognitive function^13^,^14^. Meanwhile, *Acori Graminei Rhizoma* is also frequently prescribed for the treatment of brain tumor in some Chinese medicine formulations. However, the anti-glioma effect of *Acori Graminei Rhizoma* has not been examined.

Volatile oil of *Acori Graminei Rhizoma* (VOA) has been reported to be the main bioactive components^15^. In the current study, we first determined the anti-glioma activity of VOA. Then, the role of apoptosis and autophagy played in the VOA’s anti-tumor effect was further investigated. And finally, the influence of tumor suppressor p53 on both apoptosis and autophagy triggered by VOA was specially examined.

## Results

### VOA induced differential growth inhibitory effect on p53 wild-type and p53 mutant human GBM cells

**Figure 1:**
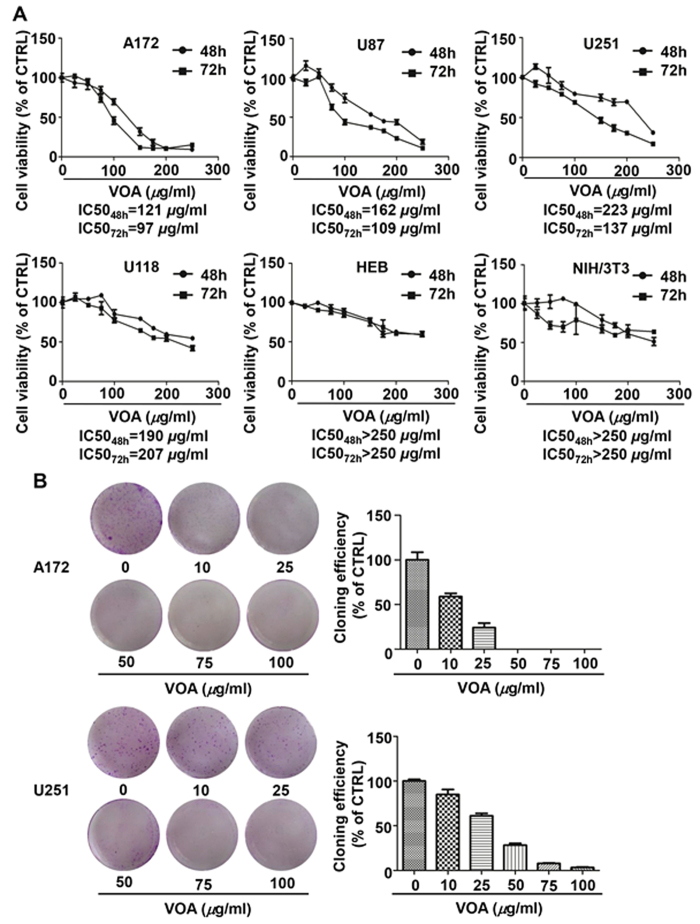
VOA induced differential cell death in human glioma cells and normal cells. (**A**) p53 wild-type cells (A172 and U87), p53 mutant cells (U251 and U118) and normal cells (HEB and NIH/3T3) were treated with 0, 25, 50, 75, 100, 150, 175, 200 and 250 *μ*g/ml VOA for 48 or 72 h and cell viability were determined by SRB assay. (**B**) Colonies in A172 and U251 cells were treated with indicated concentrations of VOA and allowed to grow for 2 weeks before stained with 0.005% crystal violet. Adjacent picture depicts the crystal violet-stained colonies and bar graph indicated the cloning efficiency compared with untreated control. Values represent mean ± SD. *p < 0.05 compared with control.

The cytotoxic effect of VOA with different concentrations on GBM cells including A172, U87, U251 and U118 cells were determined at 48 h and 72 h by Sulforhodamine B (SRB) assay. In addition, NIH/3T3 embryonic fibroblast cells and human glia HEB cells were also used to determine the cytotoxic effect of VOA in normal cells. As shown in Fig. 1A, it’s interesting to find that A172 and U87 cells, which are p53 wild-type cell lines, were more sensitive to VOA’s cytotoxicity than U251 and U118 cells, which are p53 mutant cell lines. Additionally, VOA treatment induced relatively low cytotoxicity in NIH/3T3 cells and HEB cells compared to that in human GBM cells. Furthermore, the clonogenic assay performed with a sustained treatment of A172 and U251 cells for two weeks also showed that the inhibition concentrations of VOA in A172 cells were significantly lower than that in U251 cells (Fig. 1B).

### VOA induced apoptosis in both A172 cells and U251 cells

**Figure 2:**
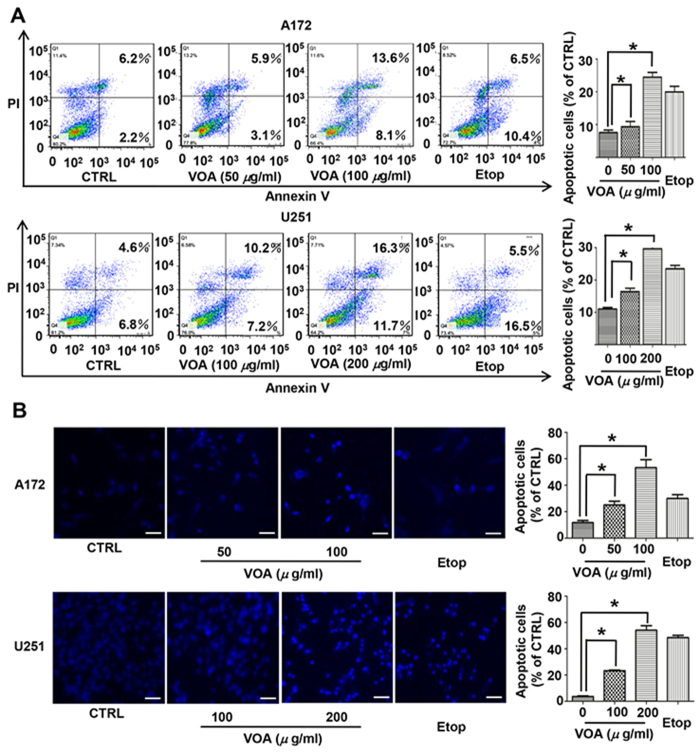
Apoptosis occurred in both A172 and U251 cells after VOA treatment. A172 and U251 cells were treated with indicated concentrations of VOA for 48 h. (**A**) Apoptotic cells were quantified by flow cytometry after stained with FITC-conjugated Annexin V and PI. (**B**) Morphologic change of apoptotic cells was evaluated by Hoechst 33342 staining. Etoposide (25 *μ*M) was used as positive control. The scale bar is 50 *μ*m. Values represent mean ± SD. *p < 0.05.

To identify whether VOA-induced cytotoxicity was due to apoptosis, we evaluated the inhibitory effect of VOA with Annexin V/PI staining analysis by flow cytometry. The result in Fig. 2A showed that a significant increase of apoptotic cells was induced by VOA treatment after 48 h in both A172 and U251 cells. In particular, the percentage of apoptotic cells increased from 8.4% to 21.7% in 100 *μ*M of VOA treated-A172 cells, while from 11.4% to 28.0% in 200 *μ*M of VOA treated-U251 cells. Furthermore, we used Hoechst 33342 staining to detect the morphologic change of apoptotic cells. As shown in Fig. 2B, both VOA treated-A172 and U251 cells exhibited much more cells with condensed and fragmented nuclei than those treated with vehicle alone.

### VOA triggered caspase-dependent apoptosis in A172 cells but caspase-independent apoptosis in U251 cells

**Figure 3:**
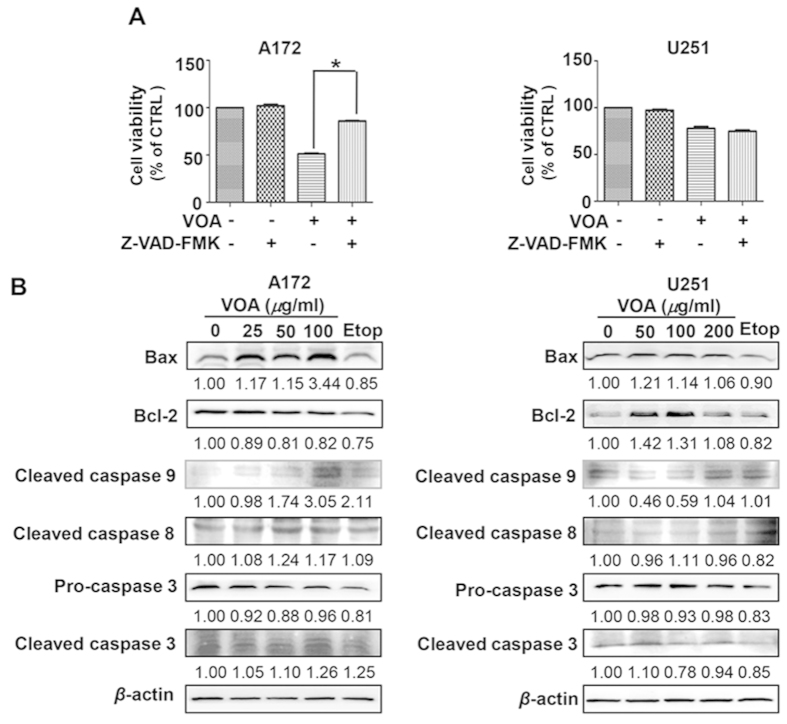
VOA induced caspase-dependent apoptosis in A172 cells and caspase-independent apoptosis in U251 cells. (**A**) Cells were pretreated with pan-caspase inhibitor Z-VAD-FMK (25 *μ*M) for 2 h and then treated with VOA (100 *μ*g/ml for A172 cells and 200 *μ*g/ml for U251 cells) for 48 h. The cell viability was determined by SRB assay. Values represent mean ± SD. *p < 0.05. (**B**) A172 and U251 cells were treated with indicated concentrations of VOA for 48 h. Then, expression of apoptosis-related proteins including Bax, Bcl-2, pro-caspase 3, cleaved caspase 3, 8 and 9 was determined by Western blotting. The blots were a representative of three independent experiments.

To further determine the potential signaling pathway involved in the VOA-induced apoptosis of A172 and U251 cells, cell viability was checked by SRB assay after pre-treatment with pan-caspase inhibitor Z-VAD-FMK^16^. As shown in Fig. 3A, exposure of A172 cells to VOA (100 *μ*g/ml) with Z-VAD-FMK mostly prevented VOA-provoked cell death while this phenomenon did not happen in 200 *μ*g/ml of VOA-treated U251 cells. These results suggest that VOA may trigger caspase-dependent apoptosis in A172 but caspase-independent apoptosis in U251 cells. To further confirm our deduction, caspase-related apoptotic proteins were determined. Western blotting analysis results in Fig. 3B revealed that VOA induced a concentration-dependent apoptosis way in A172 cells with the cleavage of caspase 3, caspase 8 and caspase 9. Meanwhile, the ratio of Bax/Bcl-2 also increased after VOA treatment for 48 h. However, neither cleavage of caspase-related proteins nor ratio of Bax/Bcl-2 significantly increased in U251 cells. These data indicate that caspase 3-mediated intrinsic and extrinsic apoptosis exists in VOA-treated A172 cells, while VOA-treated U251 cells may go to apoptosis in other ways which are still waiting to explore.

### VOA activated autophagy process in both A172 and U251 cells

**Figure 4:**
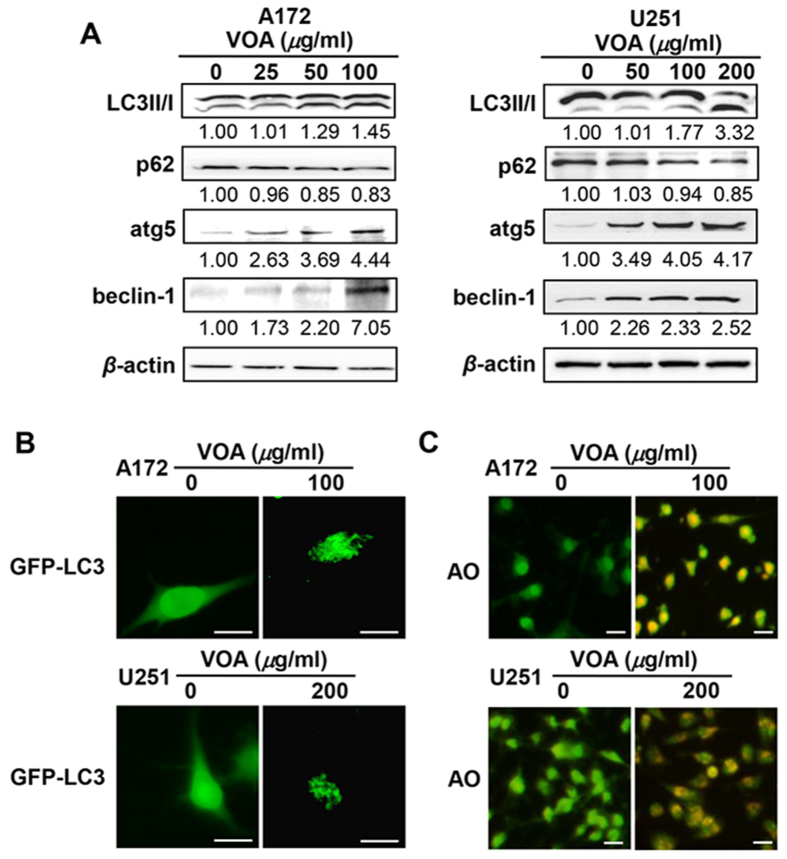
Autophagy was triggered in both A172 and U251 cells by VOA. (**A**) A172 and U251 cells were treated with indicated concentrations of VOA for 48 h. Then, expression of autophagy related proteins including LC3II/I, p62, atg 5 and beclin 1 was detected by Western blotting. The blots were a representative of three independent experiments. (**B**) A172 and U251 cells were transfected with GFP-LC3 for 6 h and then treated with VOA (100 *μ*g/ml for A172 cells and 200 *μ*g/ml for U251 cells) for 48 h. A punctate distribution of LC3 in both cells was observed by fluorescence microscope (400×). The scale of bar is 100 *μ*m. (**C**) A172 and U251 cells were treated with indicated concentrations of VOA for 48 h, then stained by acridine orange and observed under fluorescence microscope (200×). The scale of bar is 100 *μ*m.

To determine whether autophagy is involved in VOA-induced cell death, we firstly analyzed the effect of VOA on LC3-II/I protein, which is considered as a determinate maker of autophagy activation. The results (Fig. 4A) showed that VOA increased the ratio of LC3-II/I in both A172 and U251 cells. To further ascertain the autophagosome formation in VOA-treated cells, the changes of the following major autophagy related makers, p62, beclin-1 and atg5, were analyzed by Western blotting after 48 h treatment. As a result, p62 was significantly decreased in a concentration-dependent way, while atg5 and beclin1 were remarkably increased. Then, we further transfected GFP-LC3 into both A172 and U251 cells. As shown in Fig. 4B, a diffuse localization of LC3 fluorescence was observed in both untreated A172 and U251 cells, whereas a punctate pattern of LC3 fluorescence was detected in both VOA-treated cells. Further acridine orange staining (Fig. 4C) showed that there was obvious formation of acidic vesicular organelles (AVOs) in VOA-treated A172 (100 *μ*M) and U251 (200 *μ*M) cells. Together, these data strongly indicate that VOA induced autophagy in both A172 and U251 cells.

### VOA-mediated AMPK/mTOR signaling pathway in A172 cells and AMPK/mTOR-independent pathway in U251 cells

**Figure 5:**
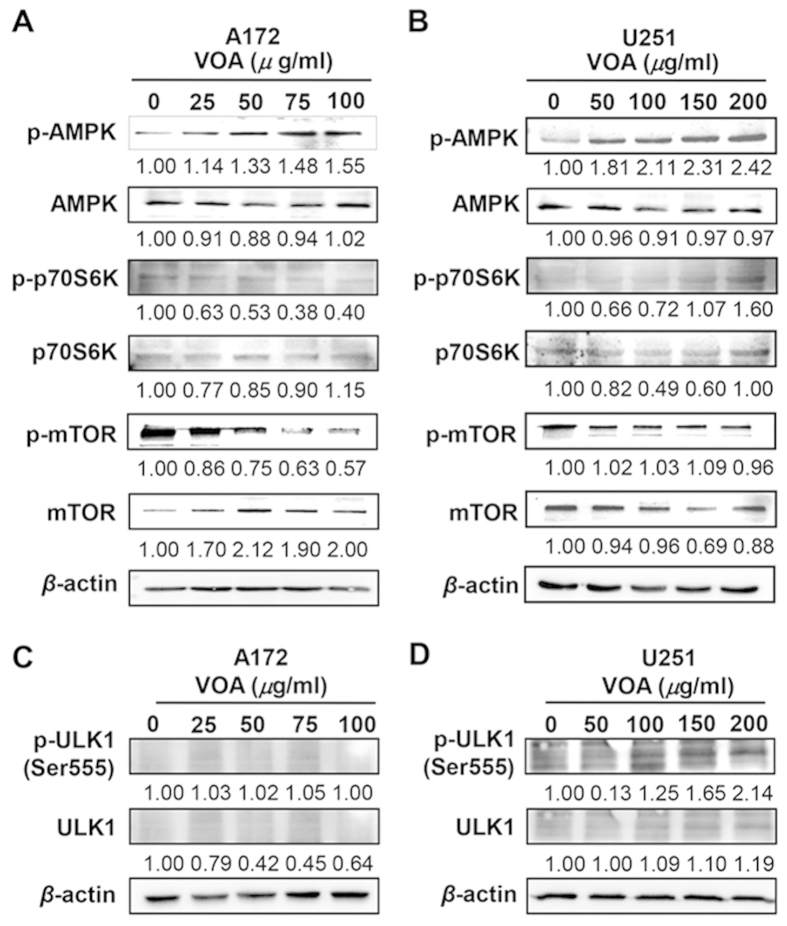
VOA induced AMPK/mTOR-dependent autophagy in A172 cells and AMPK/mTOR-independent autophagy in U251 cells. A172 (**A**,**C**) and U251 (**B**,**D**) cells were treated with indicated concentrations of VOA for 48 h. Then, expression of p-AMPK, AMPK, p-mTOR, mTOR, p-p70S6K, p70S6K, p-ULK1-Ser555 and ULK1 was detected by Western blotting. The blots were a representative of three independent experiments.

To elucidate the potential molecular mechanisms leading to the VOA-induced autophagy, we evaluated whether AMPK and mTOR were involved in this autophagy flux in both A172 and U251 cells. Our results (Fig. 5A) showed that VOA enhanced AMPK phosphorylation in A172 cells. Moreover, it inhibited the activation of the AMPK downstream targets mTOR and p70 S6K by decreasing their phosphorylation, suggesting the AMPK/mTOR signaling pathway may contribute to the activation of autophagy by VOA in A172 cells. However, we found that VOA could induce autophagy via the activation of AMPK pathway without inhibiting either phosphorylation of mTOR or its substrates p70 S6K in U251 cells (Fig. 5B). This indicated that a bypassing mechanism of AMPK signaling pathway independent of mTOR suppression may be involved in the VOA’s activation of autophagy in U251 cells. ULK1 protein kinase is a key initiator of the autophagic process and plays a critical role in the AMPK and mTORC1 mediated autophagy regulation^17^,^18^. Thus, we determined the effect of VOA on ULK1-Ser555 in A172 and U251 cells. As a result, ULK1-Ser555 was activated in U251 cells but not in A172 cells (Fig. 5C).

Recent reports also showed autophagy depends on the activation of MAP kinases signaling pathway^19^. To investigate whether the activation of MAP kinases signaling pathway contributes to the induction of autophagy in U251 cells, the expression of the phosphorylated forms of ERK1/2, P38 MAPK and JNK were examined by immunoblotting. As shown in Figure S3, VOA induced a significant activation of ERK1/2, P38 MAPK and JNK in a concentration-dependent way in U251 cells. Thus, it is possible that VOA-induced autophagy in U251 cells may also be regulated by MAP kinase signaling pathway.

### VOA-induced apoptosis and autophagy related to the decrease of p53 level in A172 cells

**Figure 6:**
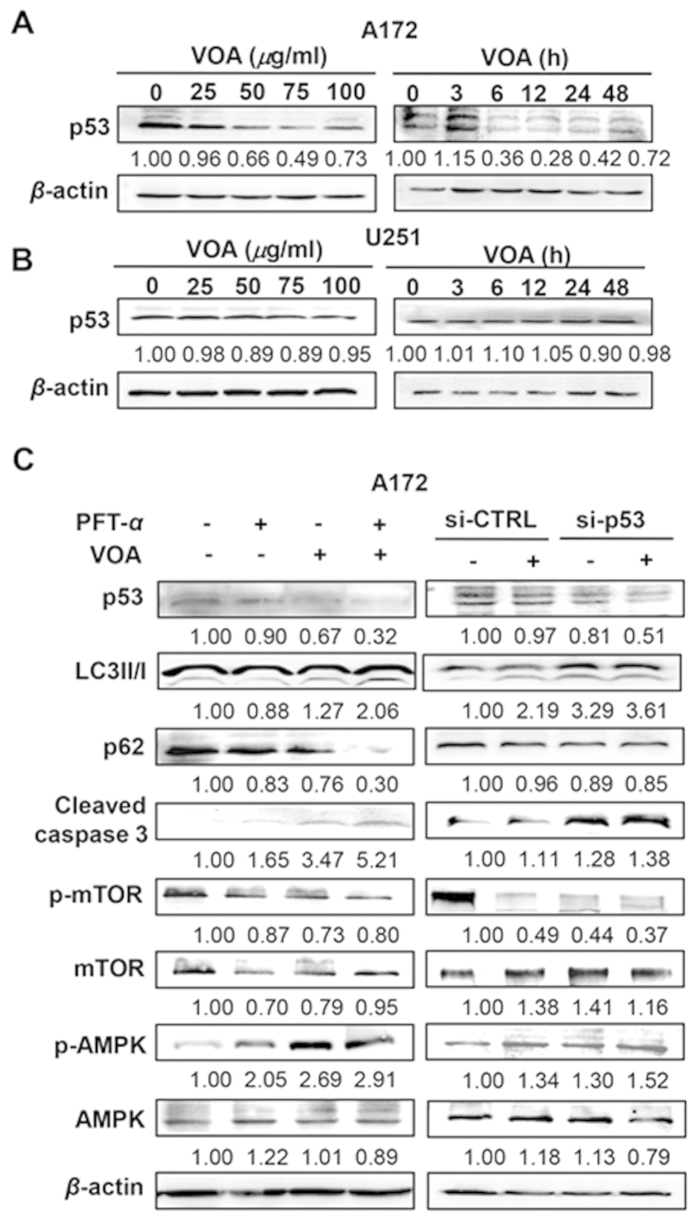
VOA-induced apoptosis and autophagy in A172 cells were associated with the decrease in p53 level. A172 (**A**) and U251 (**B**) cells were treated with indicated concentrations of VOA for 48 h or with VOA (100 *μ*g/ml for A172 cells and 200 *μ*g/ml for U251 cells) for indicated time points. Then, expression of p53 was detected by Western blotting. The blots were a representative of three independent experiments. (**C**) A172 cells were pre-treated with or without PFT-*α* (10 *μ*M) for 2 h or transfected with si-p53 or si-CTRL for 6 h, and then treated with VOA (100 *μg*/ml) for 48 h. Expression of LC3II/I, p62, cleaved caspase 3, p-mTOR, mTOR, p-AMPK, AMPK. The blots were a representative of three independent experiments.

The human tumor suppressor protein p53, which transactivates many of pro-apoptotic and cell cycle arresting-related genes, is well recognized for its ability to induce apoptosis^20^. Recent reports have also suggested a novel function of p53 in regulating autophagy^21^,^22^,^23^. Thus, to investigate the role of p53 in VOA-triggered apoptosis and autophagy, we first determined p53 expression level after VOA treatment in both A172 and U251 cells. As a result, p53 level was negatively correlated with the concentration of VOA over the range of 0–100 *μ*g/ml after 48 h treatment in A172 cells. Moreover, VOA decreased p53 expression level starting from 6 h treatment in A172 cells (Fig. 6A). This result was also confirmed by the observation that a concentration- and time-dependent p53 decrease occurred in U87 cells after VOA treatment (Figure S4A). In contrast, no p53 change was observed after VOA treatment with different concentrations and time points in U251 cells (Fig. 6B). The results implicated that VOA may reduce p53 level in p53 wild-type GBM cells compared with no total p53 alteration in p53 mutant GBM cells. Previous reports showed that *β*-asarone and *β*-elemene, two main components of VOA, exhibited p53 inducing effect^24^,^25^. Then, we determined the effect of VOA and these main components on p53 expression side by side in A172 cells. As shown in Figure S5, VOA decreased p53 expression as determined previously. Beta-asarone (25 and 50 *μ*M) and *β*-elemene (25 *μ*M) alone significantly increased p53 expression and *β*-elemene (50 *μ*M) alone exhibited no obvious effect on p53 expression. However, the combination of *β*-asarone (25 *μ*M) and *β*-elemene (25 *μ*M) remarkably reversed the up-regulation of p53 expression by these two compounds alone. Moreover, the combination of *β*-asarone (50 *μ*M) and *β*-elemene (50 *μ*M) even slightly down-regulated p53 expression compared to control. These results indicated that the effect of main components in VOA on p53 expression is concentration-dependent. When these components used as a high concentration alone or as combination, p53 expression may be reversed or even down-regulated just as the effect exhibited by VOA.

To further determine the role of p53 down-regulation after VOA treatment in A172 cells, we inhibited p53 level with pifithrin-*α* (PFT-*α*), which was validated by the inhibition of p53 responsive gene p21 expression (Figure S6) or knocked it down with a specific p53 small interfering RNA (si-p53). Figure 6C showed that VOA-induced cleaved caspase 3 and LC3II/I formation further increased, while VOA-down-regulated p62 expression further reduced after PFT-*α* pre-treatment or si-p53 transfection. These results indicated that there is a direct impact of p53 decrease on VOA-induced apoptotic and autophagic processes. In addition, it is reported that p53 mediates autophagy through an AMPK/mTOR-dependent pathway^26^, leading us to hypothesize that AMPK/mTOR may be involved in p53 loss-activated autophagy in VOA treated-A172 cells. As showed in Fig. 6B, VOA induced-decrease of p-mTOR and increase of p-AMPK were remarkably augmented after PFT-α pretreatment or si-p53 transfection in A172 cells. Furthermore, we confirmed the above observation in U87 cells. The result showed that VOA induced-increase of LC3II/I, cleaved caspase 3 and p-AMPK and decrease of p-mTOR were also augmented in U87 cells after PFT-α pretreatment (Figure S4B). Taken together, these results revealed that p53 inhibition or knockdown could enhance the induction of apoptosis and autophagy by VOA in human glioma cells harboring wild-type p53. Moreover, p53-mediated AMPK/mTOR pathway may play a critical role in VOA-induced autophagy in A172 cells.

### Inhibition of autophagy switches it to apoptosis in both A172 and U251 cells with VOA intervention

**Figure 7:**
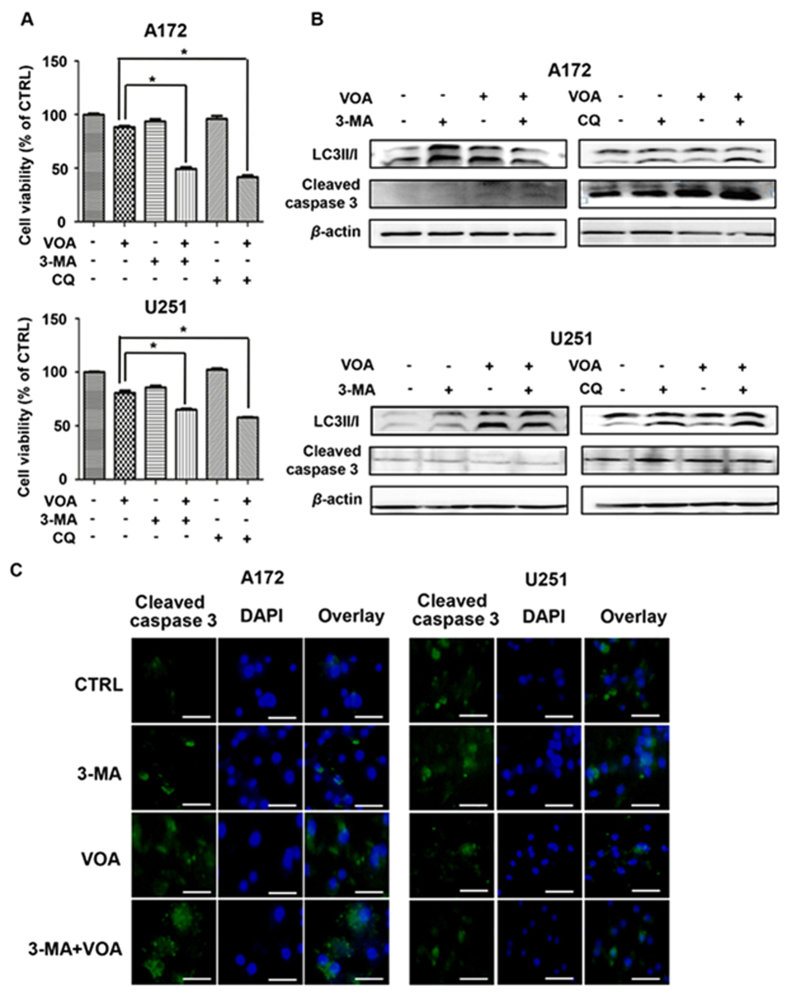
Inhibition of autophagy enhanced VOA-induced apoptosis. A172 and U251 cells were pretreated with or without 3-MA (2.5 mM) or CQ (10 *μ*M) for 2 h and then treated with VOA (100 *μ*g/ml) for 48 h. (**A**) Cell viability of A172 and U251 cells were measured by SRB assay. Values represent mean ± SD. *p < 0.05. (**B**) Expression of LC3II/I and cleaved caspase 3 was detected by Western blotting. The blots were a representative of three independent experiments. (**C**) A172 and U251 cells were pretreated with or without 3-MA (2.5 mM) for 2 h and then treated with VOA (100 *μ*g/ml) for 48 h. Immunofluorescence staining of cleaved caspase 3 was observed by fluorescence microscope (400×). The scale of bar is 100 *μ*m.

There is a complex relationship between autophagy and apoptosis in the modulation of cellular survival responses^22^,^23^,^27^. To investigate the role of autophagy in VOA-induced cell death, we used 3-methyladenine (3-MA) or chloroquine (CQ) to inhibit the autophagy caused by VOA. The results of SRB assay showed in Fig. 7A demonstrated that inhibition of autophagy by both 3-MA and CQ is capable of promoting the VOA-induced cell death in both A172 and U251 cells. Next, Western blotting analysis showed that co-treatment VOA with 3-MA or CQ caused an increase of caspase 3 cleavage in A172 cells (Fig. 7B), suggesting that inhibition of autophagy promotes the caspase-dependent apoptosis in p53 wild-type cells. However, there is no change of cleaved caspase 3 in U251 cells, further suggesting that a caspase-independent apoptosis is triggered by VOA in U251 cells. These results were also confirmed by immunocytofluorescent staining. Figure 7C showed that co-treatment of VOA with 3-MA caused higher fluorescent level of activated caspase 3 in A172, while no remarkably change was observed in U251 cells. These results indicate that VOA-induced autophagy is a protective mechanism and blockage of autophagy aggravates the VOA-induced apoptosis. In Figure S7, flow cytometry analysis showed that apoptotic cell death increased from 20.1% to 30.4% in 100 *μ*g/ml of VOA-treated A172 cells and from 19.8% to 31.0% in 200 *μ*g/ml of VOA-treated U251 cells after the incorporation of 3-MA. However, 3-MA alone in both A172 and U251 also led to increased apoptotic cells compare to control. Therefore, based on this result, the potential role of 3-MA-induced apoptosis couldn’t be excluded from the apoptosis inducing effect of VOA and 3-MA co-treatment.

## Discussion

Despite considerable advances in brain cancer therapy in the past decade, the prognosis for patients having glioblastoma remains extremely poor^28^,^29^. The combined treatment based on radiotherapy and temozolomide is considered to be the optimal treatment for patients with glioblastoma, which doubles the 2-year survival rate to 27%. However, the efficacy of temozolomide is limited due to its drug resistance and toxic side effects^30^,^31^. Thus, it is crucial to develop new chemotherapeutic agents for treating patients with glioblastoma.

In recent years, Chinese herbal medicine has received extensive attention as the source of new drugs for anti-cancer therapy^32^. *Acori graminei Rhizoma* is traditionally used for CNS disorders in China for more than hundreds of years. Though it is frequently prescribed in the formulations for the treatment of brain tumor, the effect is still not directly validated and the potential mechanisms are also unclear. The volatile oil of *Acori graminei Rhizoma* (VOA) is believed to be the main active components. The major compounds existing in the VOA are detectable in the rat brain tissue after oral administration^33^. And importantly, they are reported to inhibit the function and expression of P-glycoprotein and improve the permeability of BBB^34^,^35^.

In the present study, we first investigated the growth inhibitory effect of VOA on different human GBM cell lines and normal cell lines. VOA exhibited low cytotoxicity to normal cell lines, while it induced significant cytotoxicity to human GBM cell lines. Interestingly, the cytotoxicity of VOA seems to be related to p53 status as p53 wild-type GBM cells (A172 and U87) are more sensitive to p53 mutant GBM cells (U251 and U118). Furthermore, the result of clonogenic assay also supported our deduction.

Natural products usually exert their anticancer effects by triggering apoptosis and autophagy^36^,^37^. Thus, we first determined the role of apoptosis in VOA induced cell death. The results of Annexin V/PI staining and Hoechst 33342 staining demonstrated that apoptosis was induced by VOA in both A172 cells and U251 cells. However, it’s interesting that pre-treatment with pan-caspase inhibitor Z-VAD-FMK mostly prevented VOA-provoked cell death in A172 cells, whereas this phenomenon did not happen in U251 cells, suggesting that caspase-dependent and independent apoptotic pathways were triggered in A172 and U251 cells, respectively. Apoptosis related proteins caspase 3, caspase 8 and caspase 9 play extremely important roles in the apoptotic signaling ways. Caspase 3, a key executioner in the apoptotic machinery, cleaves many proteins indispensable for cell survival and is activated through cleavage into two fragments (17 kDa and 19 kDa) by caspase 8 and/or caspase 9 during apoptosis^38^. Caspase 8 and caspase 9 play crucial roles in the induction of apoptosis through death receptor-mediated (extrinsic) and the mitochondrial (intrinsic) pathways, respectively^39^. Moreover, the balance between expression levels of anti-apoptotic Bcl-2 and pro-apoptotic Bax is also critical for cell survival and death^40^, where increase of the Bax/Bcl-2 ratio activates the release of cytochrome C from mitochondria and subsequently activates the intrinsic apoptotic pathway^22^. Therefore, the effect of VOA on these apoptosis related proteins was further determined in this study. Our results demonstrated that VOA obviously increased the cleavage of apoptosis-executive caspase 3, caspase 8 and caspase 9 and also contributed to a significantly increased Bax/Bcl-2 ratio, suggesting that the mitochondrion-dependent intrinsic apoptosis pathway and receptor-mediated extrinsic pathway participated in VOA associated cytotoxicity in A172 cells. However, the results obtained from U251 cells demonstrated that VOA neither promoted the cleavage of caspase family proteins, nor changed the Bax/Bcl-2 ratio. A possible explanation for this is the different genetic background of these two cell models. It is reasonable to assume that VOA-induced apoptotic signaling pathway may be dependent on p53 deficient/mutant status.

As a complementary mechanism of cell death, autophagy is also involved in the anti-cancer activity of a wide variety of natural products^41^. We then determined whether autophagy was triggered in VOA-induced cell death. Our study revealed that VOA remarkably increased the ratio of LC3-II/I, atg5 and beclin1 and decreased p62 levels in both A172 and U251 cells, indicating that autophagy was induced by VOA in these two different cell lines. This effect was further validated by observation with fluorescence microscope after GFP-LC3 transfection and AO staining, where VOA significantly increased the punctate pattern of LC3 fluorescence and the formation of AVOs. It’s well known that multiple upstream signaling pathways are involved in the induction of autophagy, including beclin-1 (atg6), AMPK/mTOR, calcium signaling, endoplasmic reticulum-stress and Ras/Raf/MAPK pathway^42^. Among them, AMPK/mTOR plays a central role in the regulation of autophagy by integrating and coordinating different sensory inputs from upstream factors and then phosphorylating Atg proteins^31^,^43^. In our study, it’s noticeable that VOA induced autophagy via AMPK/mTOR signaling pathway in A172 cells but independently of mTOR inhibition in U251 cells. Similarly, previous investigation also reported that fangchinoline induced autophagy via the activation of the AMPK pathway without inhibiting either phosphorylation of mTOR^26^. ULK1-Ser555 has been reported to be phosphorylated by AMPK directly^44^. Our further result demonstrated that VOA-induced autophagy in U251 cells may be mediated by AMPK via directly phosphorylating ULK1-Ser555, which is consistent with the result that VOA-induced autophagy is independent of mTOR in U251. Meanwhile, our result also indicated that ULK1-Ser555 was not involved in VOA-induced autophagy in A172 cells, in which the AMPK/mTOR signaling pathway played a critical role as reflected in Fig. 5A.

It has been reported that p53 was found to play critical roles in the regulation of not only apoptosis, but also autophagy^21^,^43^,^45^. In our study, VOA remarkably decreased p53 level in a concentration- and time-dependent manner in A172 and U87 cells, while no significant change was observed in U251 cells. Recent investigations revealed that p53 decrease is closely related to apoptosis and autophagy induced by different stimuli^46^. Thus, we further evaluated the role of p53 decrease in VOA-induced apoptosis and autophagy. Similarly, our results showed that augment of both apoptosis and autophagy induced by VOA could be achieved by co-treatment of p53 inhibitor PFT-α in both A172 and U87 cells or transfection of p53 siRNA in A172 cells. Meanwhile, p53 decrease further enhanced the VOA-activated AMPK/mTOR in both A172 and U87 cells, strongly suggesting that VOA induced autophagy in wild-type GBM cells by p53-mediated AMPK/mTOR pathway. These results are in accordance with previous reports that docosahexaenoic acid (DHA)^47^ and mimulone^38^ induced autophagy via p53/AMPK/mTOR signaling in human cancer cells with wild-type p53.

In many cases, apoptosis and autophagy were simultaneously induced by natural products^46^,^47^. The interconnection between them is complex and varies with the nature of stimulus and cell type. As autophagy is a process that consumes cellular components and generates energy, it may sensitize cells to apoptosis by acting as an energy source or suppress apoptosis as protective autophagy by clearing damaged organelles under stress condition^48^. In the present study, blockage of autophagy by 3-MA or CQ augmented apoptosis induced by VOA in both A172 and U251 cells, indicating that VOA-induced autophagy might play a protective role.

## Conclusion

In this study, VOA exhibited obvious growth inhibitory effect on human malignant glioma cells, which seems to be closely related to p53 status. Apoptotic cell death and protective autophagy were induced by VOA in both A172 and U251 cells. Moreover, the underlying mechanisms are cell type specific and dependent on p53 status. Further study could be ongoing to explore whether there are some ingredients in the *Acori Graminei Rhizoma*-containing formulations that regulate autophagy to sensitize tumor cells to anticancer therapy.

## Materials and Methods

### Materials

VOA was extracted from *Acori Graminei Rhizoma* by a well-established procedure in our lab (the details described in Supplementary Methods) and stored in −80 °C before use. Four major components (*β*-asarone, tatarine A, *β*-elemene and zierone) were identified by HPLC-MS in this extract (Figures S1 and S2). The antibodies against caspase 3, cleaved caspase 3, cleaved caspase 9, LC 3II/I, atg 5 and beclin-1 were obtained from Cell Signaling Technology (Boston, MA, USA). SQSTM1/p62 antibody was obtained from Santa Cruz Biotechnology (CA, USA). The antibodies against *β*-actin and rabbit IgG were obtained from Sigma-Aldrich (St. Louis, MO, USA). The antibodies against p53 and cleaved caspase 8 were obtained from Wanlei Biotechnology (Shenyang, China). Other chemicals were obtained from Sigma-Aldrich Co. (St. Louis, MO, USA) unless indicated otherwise.

### Cell culture

Human glioblastoma multiforme (GBM) cells A172, U87 and U251 and mouse embryonic fibroblast NIH/3T3 cells were obtained from Kunming Institute of Zoology, CAS. Human GBM U118 and human glial cells HEB were obtained from Zhongshan University. All the cells were maintained in Dulbecco’s modified Eagle’s medium (DMEM) supplemented with 10% fetal bovine serum (FBS) (Invitrogen, USA) and 1% penicillin/streptomycin (Invitrogen, USA) at 37 °C in a humidified 5% CO2 atmosphere.

### Measurement of cell viability

Cell viability was evaluated by sulforhodamine B (SRB) assay (Sigma-Aldrich, St. Louis, MO, USA), which was based on the measurement of cellular protein content^49^,^50^ and stained with 0.4% SRB for 30 min. The protein-bound dye is dissolved in 10 mM Tris base solution for OD determination at a wavelength of 490 nm using a multi-well spectrophotometer microplate reader (Biotek, Winooski, VT, USA). Cell viability was expressed as a percentage of that of the control (untreated) cells.

### Clonogenic assay

Cells were seeded in a 6-well plate and treated with different concentrations of VOA. Colonies were allowed to form for two weeks and medium with VOA or vehicle was replaced per 3 days. At the end of treatment, cells were fixed in 100% methanol and stained with 0.005% crystal violet. Finally, images were captured by a CCD camera and the colonies were counted.

### Flow cytometry

Cells were seeded into 6-well plates at a density of 1.6 × 10^5^ cells/well. After drug treatment, cells were harvested, washed and resuspended in the binding buffer containing annexin V and propidium iodide (PI). After incubation at room temperature in the dark for 15 min, the stained cells were subjected to a BD LSRFortessa Cell Analyzer (BD Biosciences, San Jose, CA, USA) with fluorescence emission at 530 nm and 575 nm and excitation at 488 nm. Data were analyzed using Flow Jo 7.6.1 software (Tree Star, Inc., Ashland, OR, USA).

### Hoechst 33342 staining

As previously described^51^,^52^, VOA-treated cells were stained with apoptosis-specific dye Hoechst 33342. After treatment at 6-well plates for 48 h, cells were washed with 1×PBS for 3 times. Then, Hoechst 33342 dissolving in 1×PBS was added into each well. The plates were kept at room temperature for 10 min and avoided from light. Finally, the plates were washed with 1×PBS again and images were captured on a Zeiss fluorescence microscope (Carl Zeiss, Germany).

### GFP-LC3 transfection

As previously described^53^, cells were seeded into a 6-well plate at a density of 1.0 × 10^5^ cells/well and allowed to reach approximately 40–50% confluence on the day of transfection. Cells were then transfected with 4 *μ*g plasmid of green fluorescent protein-microtubule-associated protein 1 light-chain 3 (GFP-LC3) using transfection reagent Lipofectamine 2000 (Invitrogen, USA) according to the manufacturer’s instructions. After a 6 hour medium incubation, the transfection medium was removed, and the cells were incubated in fresh medium for 24 h, followed by the treatment with different concentrations of VOA. The accumulation of intense punctuate aggregations of GFP-LC3 was observed and recorded under a Zeiss fluorescence microscope (Carl Zeiss, Germany).

### Acridine orange staining

As previously described^54^,^55^, cells were seeded into 6-well plate at a density of 1.6 × 10^5^ cells/well. After treatment with VOA, cells were washed twice with 1×PBS, followed by incubation with 0.005 *μ*g/ml acridine orange (Invitrogen, USA) at 37 °C for 15 min. The stained cells were washed with 1×PBS and the fluorescence signal was detected and recorded under a Zeiss fluorescence microscope (Carl Zeiss, Germany).

### RNA interference

Small interfering RNAs targeting p53 (si-p53) and a control siRNA (si-CTRL) were obtained from sigma (St. Louis, MO, USA). Cells were seeded into a 6-well plate at a density of 1.0 × 10^5^ cells/well and allowed to reach approximately 50% confluence on the day of transfection. Cells were then transfected with 50 nM siRNA using transfection reagent Lipofectamine 2000 (Invitrogen, USA) according to the manufacturer’s instructions. After a 6 hour antibiotic-free medium incubation, the transfection medium was removed, and the cells were incubated in fresh medium for 24 h, followed by further drug treatments.

### Western blotting analysis

The cellular proteins were extracted from A172 and U251 cells in ice-cold RIPA buffer (Cell Signaling Technologies, USA) supplemented with 1% (v/v) protein inhibitor cocktail and 1 mM phenylmethylsulfonyl fluoride (PMSF). Thirty micrograms of the cellular proteins were resolved by electrophoresis in 12% SDS-polyacrylamide gel, and subsequently transferred to polyvinylidene difluoride (PVDF) membrane. Following 1 h incubation in a fresh TBS buffer containing 0.1% Tween-20 and 5% BSA, the blots were probed with specific primary antibodies. After incubation with the relevant secondary antibodies, the reactive bands were identified using an enhanced chemiluminescence (ECL) detection reagent (GE Healthcare, Sweden). The concentration of the loaded cellular proteins was normalized against the internal control *β*-actin, and then the value was expressed as each normalized data relative to control.

### Statistical analysis

All data were presented as mean ± SD for three independent experiments. Statistical analysis was performed by two-tail Student’s t-test. A p-value of less than 0.05 was considered to be statistically significant.

## Additional Information

**How to cite this article**: Chen, L. *et al.* Volatile Oil of *Acori Graminei Rhizoma*-Induced Apoptosis and Autophagy are dependent on p53 Status in Human Glioma Cells. *Sci. Rep.* **6**, 21148; doi: 10.1038/srep21148 (2016).

## Supplementary Material

Supplementary Information
