# Supplementary material for: Volatile Oil of Acori Graminei Rhizoma-Induced Apoptosis and Autophagy are dependent on p53 Status in Human Glioma Cells
Source: Sci Rep. 2016 Feb 19;6:21148. doi: 10.1038/srep21148 (PMC4759692; doi:10.1038/srep21148)
Supplement: Supplementary Information [file srep21148-s1.doc]

**Supplementary Information**

**Volatile Oil of *Acori Graminei Rhizoma*-Induced Apoptosis and Autophagy are dependent on p53 Status in Human Glioma Cells**

Lu Chen, Zhuyun Jiang, Hui Ma, Ning Ling, Hongdan Chen, Li Li*, Hongyi Qi*

-Supplementary Methods

-Supplementary Figure S1

-Supplementary Figure S2

-Supplementary Figure S3

-Supplementary Figure S4

-Supplementary Figure S5

-Supplementary Figure S6

-Supplementary Figure S7

**Supplementary Methods**

**Extraction of VOA from *Acori Graminei Rhizoma***

*Acori Graminei Rhizoma* was purchased from Sichuan Neautus Traditional Chinese Medicine Co., Ltd., which is a GMP certified pharmaceutical producer of herbal slices in China. The voucher specimens are deposited in our laboratory. The granule size of *Acori Graminei Rhizoma* material (1kg) is 60-20 mu. VOA was extracted using the supercritical-CO_2_ fluid system (Huaan, Jiangsu, China). The extraction temperature is 40 °C and the extraction pressure is 30.5 MPa. The extraction time is 2 h. Finally, the above material yields an amount of VOA 20.16 g. The extract was stored at -80 °C before use.

**Analysis of VOA by HPLC-MS/MS**

To control the quality of VOA, we have analyzed the major constituents by HPLC-MS/MS system consisting of an API 2000 Qtrap hydrid QqQ-linear ion-trap mass spectrometer equipped with TurboIonSprayTM source (Applied Biosystems, Foster City, CA, USA) and HP 1100 series HPLC system (Agilent Technologies, Palo Alto, CA, USA) equipped with a XTerraTM Ms C18 column (3.5*μ*m, 2.1mm×150mm; Waters, Milford, MA, USA). Chromatographic separation was carried out using a gradient of 10 mM ammonium acetate buffer containing 0.1 % (v/v) formic acid (A) and acetonitrile with 0.1 % (v/v) formic acid (B). The flow rate was kept at 1.4 mL/min, and the sample volume injected was set at 5 μL. Separation of analytes was obtained in 10 min with Quantitation on the LC-MS/MS system of each protonated species.

**Figures:**

**Figure S1**

**
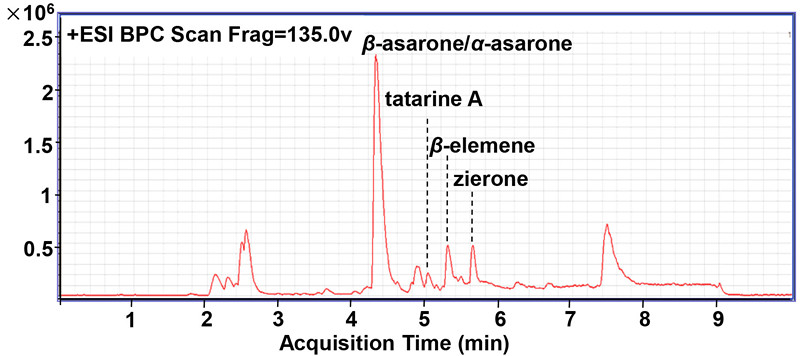
**

**Figure S1. Representative HPLC chromatogram of VOA.** Four peaks with retention times of 4.3, 5.0, 5.3 and 5.6 min are identified as *β/α*-asarone, tatarine A, *β*-elemene and zierone, respectively.

**Figure S2**

**
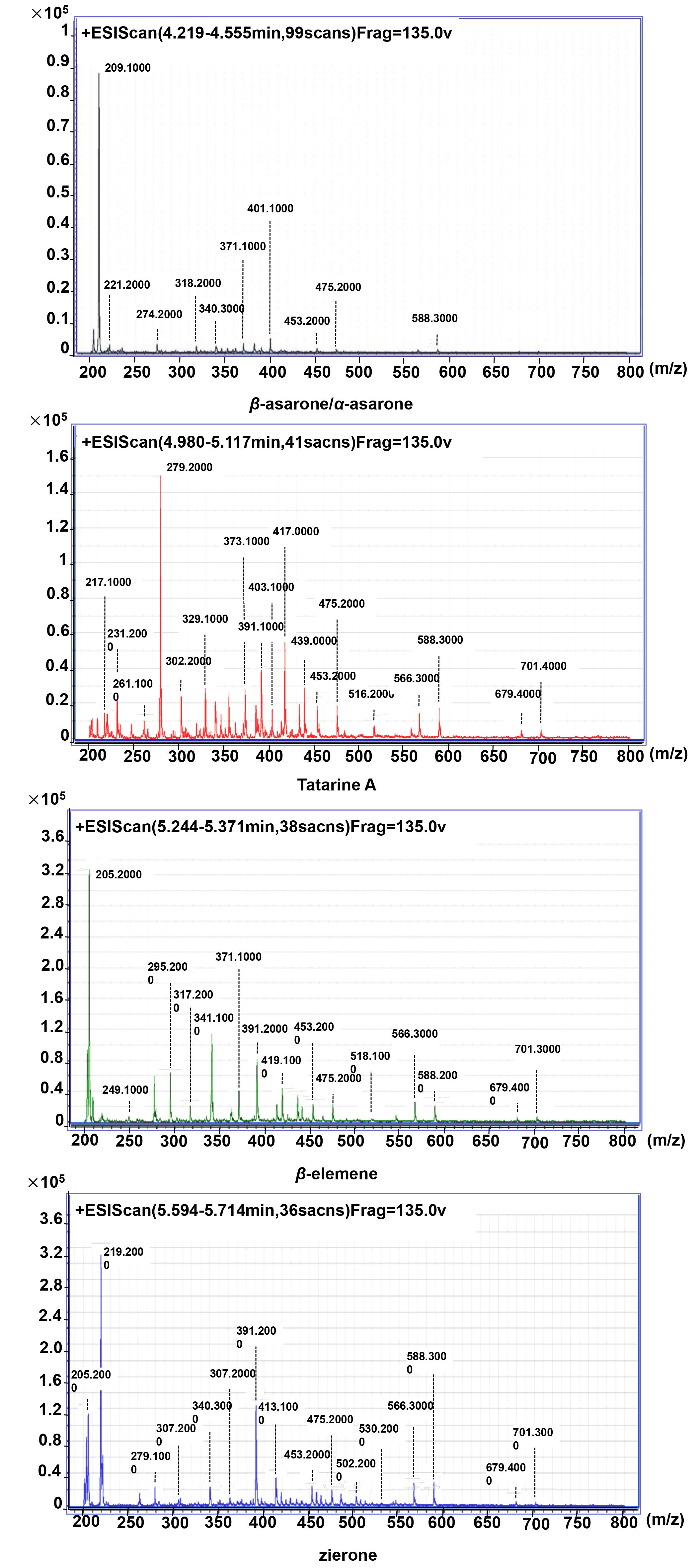
**

**Figure S2. Mass spectrogram of *β/α*-asarone, tatarine A, *β*-elemene and zierone.**

**Figure S3**

**
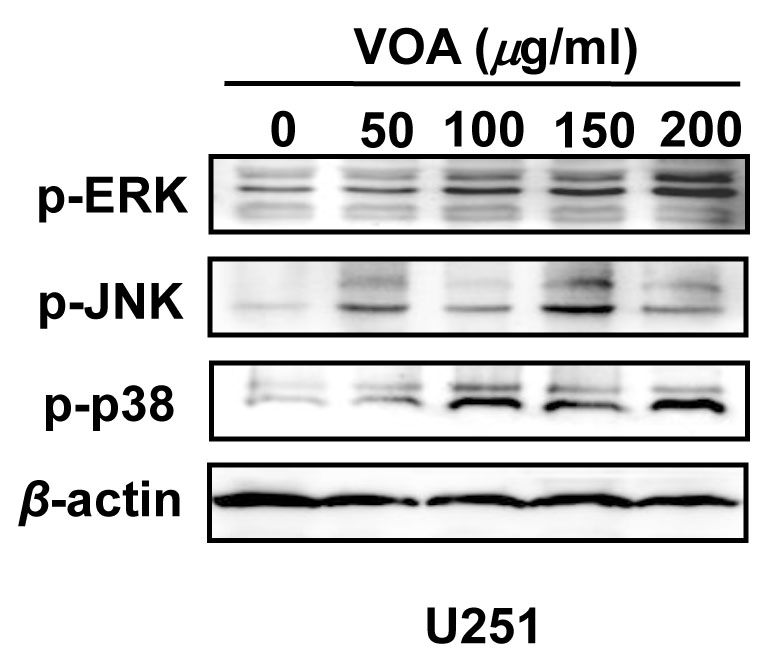
**

**Figure S3. VOA induced MAP Kinase signaling pathway.** U251 cells were treated with VOA (0, 50, 100, 150, 200 *μ*g/ml) for 48 h. Then, p-JNK, p-p38 and p-ERK were detected by immunoblotting and the results were a representative of three independent experiments.

**Figure S4**

**
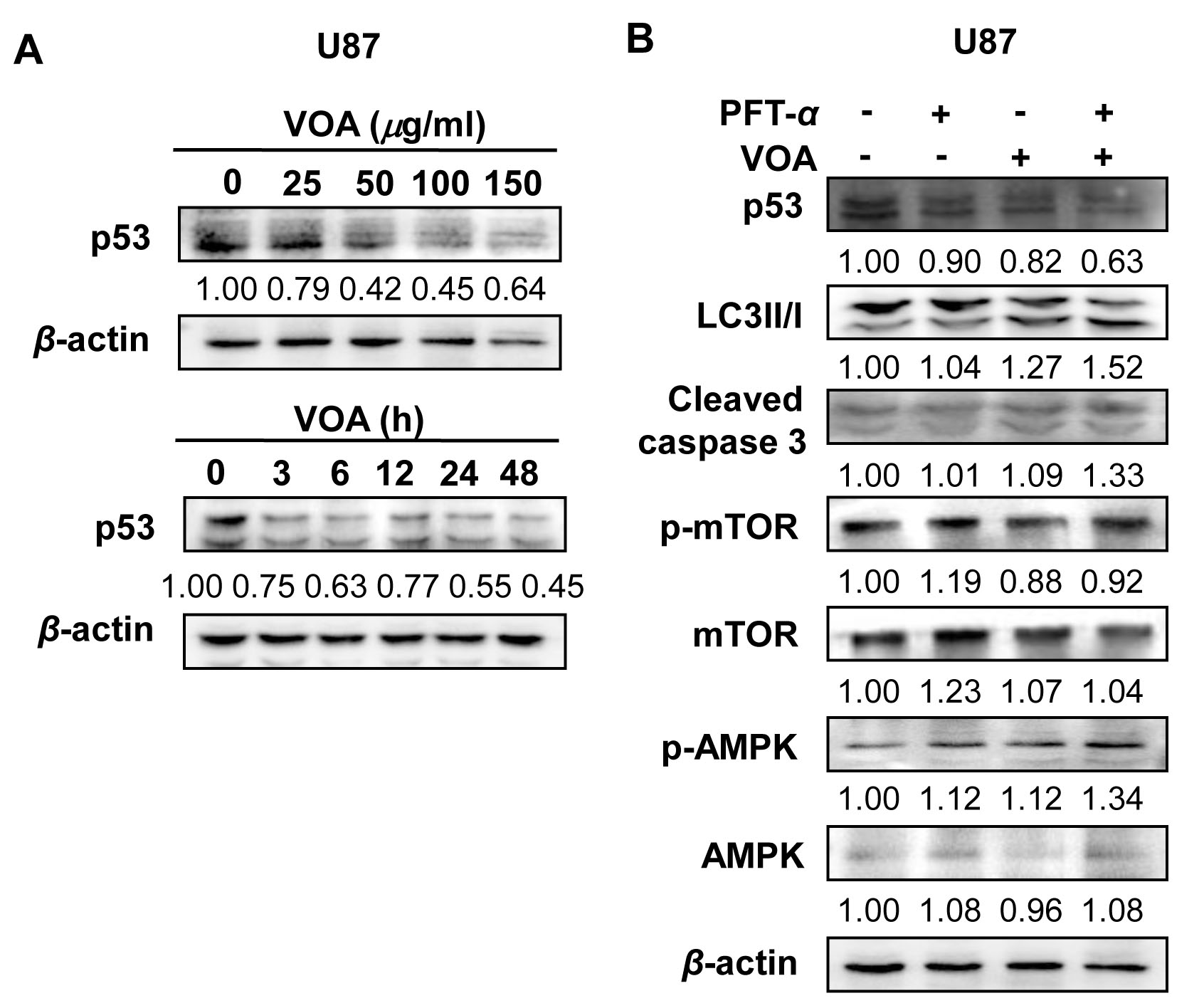
**

**Figure S4. VOA-induced apoptosis and autophagy in U87 cells were associated with the decrease in p53 level.** (A): The expression of p53 was detected in concentration and time-dependent way in U87 cells by Western blotting. The blots were a representative of three independent experiments. (B): U87 cells were pre-treated with or without PFT-*α* (10 *μ*M) for 2 h, then it was treated with VOA (150 *μg*/ml) for 48 h. Expression of p53, LC3II/I, cleaved caspase 3, p-mTOR, mTOR, p-AMPK and AMPK was detected by Western blotting. The blots were a representative of three independent experiments.

**Figure S5**

**
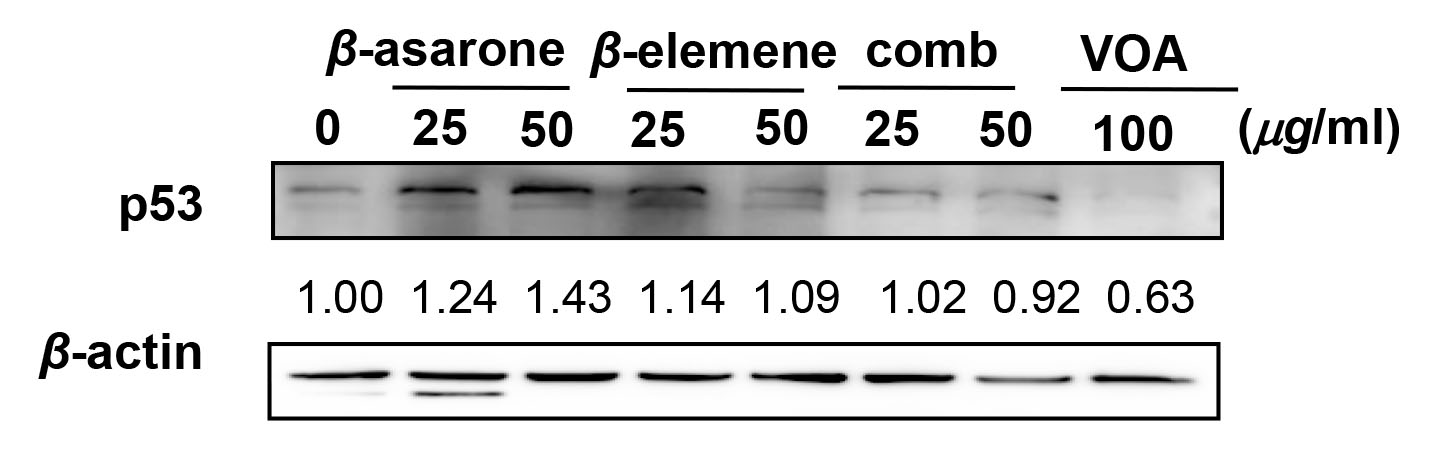
**

**Figure S5. Effect of *β*-asarone, *β*-elemene and their combination on p53 expression in A172 cells.** A172 Cells were treated with VOA, *β*-asarone, *β*-elemene or their combination (comb) with indicated concentration for 48h. The expression of p53 was detected by Western blotting. The blots were a representative of three independent experiments.

**Figure S6**

**
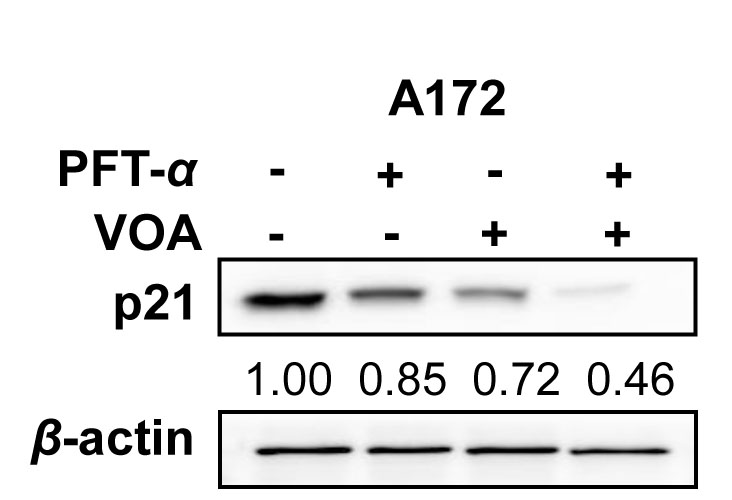
**

**Figure S6. Effect of PFT-α and VOA on p53 responsive gene p21.** (A): A172 cells were pre-treated with or without PFT-*α* (10 *μ*M) for 2 h, then p53 responsive gene p21 was detected after 48 h treatment of VOA. The expression of p21 was detected by Western blotting. The blots were a representative of three independent experiments.

**Figure S7**

**
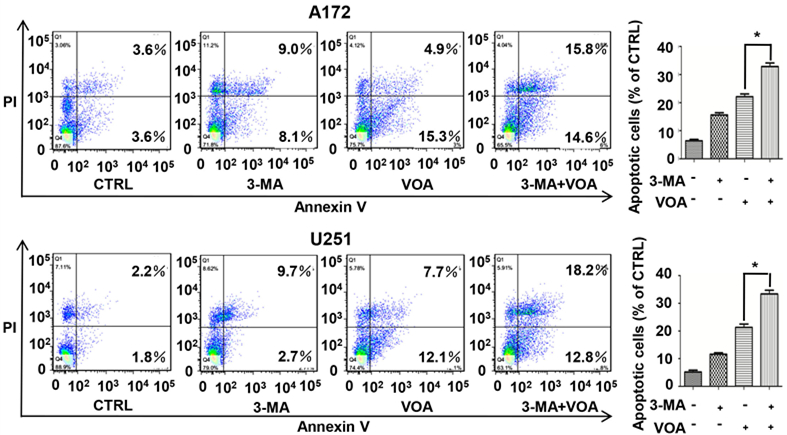
**

**Figure S7. Inhibition of autophagy enhanced VOA-induced apoptosis in both cells.** A172 was pre-treated with or without 3-MA (2.5 mM) for 2 h and then treated with VOA (100 *μ*g/ml) for 48 h. Cells were stained with FITC-conjugated Annexin V/PI and then measured by flow cytometry. Values represent mean±SD. **p*<0.05.
